# No Evidence for *Wolbachia* Effects on the Thermal Preference of the Invasive Pest *Liriomyza huidobrensis*

**DOI:** 10.3390/insects15100784

**Published:** 2024-10-09

**Authors:** Yuxi Zhu, Xinyu Wang, Sibo Wang, Zhangrong Song, Yuzhou Du

**Affiliations:** 1 Department of Entomology, College of Plant Protection, Yangzhou University, Yangzhou 225009, China; yuxizhu@yzu.edu.cn (Y.Z.); mx120220834@stu.yzu.edu.cn (X.W.); 201805222@stu.yzu.edu.cn (S.W.); 2 Entomology and Nematology Department, University of Florida, Gainesville, FL 32611, USA; zhangron.song@ufl.edu

**Keywords:** *Wolbachia*, leaf miner, *Liriomyza huidobrensis*, thermal preference, behavior

## Abstract

**Simple Summary:**

Maternally transmitted *Wolbachia* is one of the most common endosymbionts in many arthropods, influencing various aspects of host physiology, reproduction, and fitness. While recent studies on *Wolbachia*-*Drosophila* systems have revealed its influence on host thermal choice behavior, its broader impact on host thermal preference (Tp) in non-model insects remains poorly understood. The polyphagous leaf-miner fly, *Liriomyza huidobrensis* (Blanchard), one of the most notorious pests of vegetable and flowers globally, harbors a range of bacterial symbionts, with *Wolbachia* being especially prevalent. This study aims to explore the effects of *Wolbachia* on the thermal preference of the invasive leaf-miner *L. huidobrensis*. Understanding the potential roles of *Wolbachia* in host thermal behavior is crucial for elucidating the co-evolution of invasive species and their endosymbiont *Wolbachia* in the context of global climate change and temperature extremes, and may offer insight for the development of pest control strategies utilizing *Wolbachia*.

**Abstract:**

Heritable endosymbiont *Wolbachia* is prevalent among arthropods, serving multiple functions for their hosts. However, the role of *Wolbachia* in mediating thermal preference selection remains largely unexplored. In this study, we utilized a custom-built thermal gradient to evaluate the thermal preference (Tp) of 1367 individuals of the invasive leaf-miner *Liriomyza huidobrensis* with or without *Wolbachia* wLhui from Yunnan and Xinjiang populations. Under meticulously controlled conditions and with a vast sample size, we found no significant difference in the mean Tp between wLhui-infected and uninfected leaf miners from either population when host age and sex were not considered. Furthermore, generalized linear model (GLM) analysis revealed no significant correlation between average Tp and age, sex, or *Wolbachia* infection, nor interactions among these factors, except in the Xinjiang population, where Tp was strongly associated with host age. Finally, we discuss the ecological implications of these findings and propose future research directions on *Wolbachia*-mediated host Tp in the leaf miner. Overall, our findings do not provide evidence that *Wolbachia* significantly affects the thermal preference of *L. huidobrensis*. Further studies across different systems are needed to investigate the complex interactions between *Wolbachia* and insect thermal behavior.

## 1. Introduction

Maternally inherited *Wolbachia* is one of the most widespread intracellular endosymbionts in arthropods, infecting over 50% of insect species as well as numerous species of spider mites [1,2,3,4]. *Wolbachia* has wide-ranging effects on the ecology and evolution of its insect hosts, including reproductive manipulations [5,6], nutrient provision [7,8,9], thermotolerance modification [10,11], and pathogen resistance [12,13]. As our understanding of *Wolbachia*’s effects on the host phenotype grows, the development of *Wolbachia*-based strategies for the control of agricultural pests has made great progress [14,15]. Recent studies suggest *Wolbachia* can manipulate host behavior to mitigate environmental stress, which is crucial for maintaining stable symbiosis [10,16]. However, the strategies of employing *Wolbachia* to regulate host thermal preference under stress remain poorly understood.

Until recently, only a few studies on model *Drosophila* flies and non-model spider mites have demonstrated *Wolbachia*’s ability to influence host thermal preference behavior [17,18]. Our previous research showed that *Wolbachia*-infected spider mites *Tetranychus truncatus* preferred lower temperatures [18], consistent with findings in flies infected with *Wolbachia* strains such as *w*Mel, *w*MelCS, and *w*MelPop [17,19,20]. However, comparative studies in flies yielded conflicting results, with *w*Mau-infected flies favoring warmer temperatures [17], and some studies failing to identify significant impacts [21]. These variable effects suggest that *Wolbachia*-induced thermal preference may depend on host genetics, specific *Wolbachia* strains, and environmental factors [16,20], raising questions and generating debate about the influence of maternally inherited endosymbionts on insect thermal preference.

The invasive leaf-miner *Liriomyza huidobrensis*, a notorious pest of vegetable and flowers, has spread globally, causing substantial economic losses [22,23]. The *Liriomyza huidobrensis* was first reported in Yunnan, China in 1993 [24]. This pest species prefers cool environments and has rapidly expanded into multiple cool regions of China, including Xinjiang [24,25]. Our previous survey revealed that the endosymbiont *Wolbachia* is widespread in natural populations of the leaf-miner *L. huidobrensis* [26,27]. Since heritable symbionts in invasive insects may influence host adaption and expansion in various ways, including modulating host thermal preference [19,20,28], we hypothesize that the endosymbiont *Wolbachia* could manipulate host thermal preference, helping the insect cope with temperature stress during invasions.

To test the hypothesis, we assessed the thermal preference of *Wolbachia*-infected and uninfected leaf miners using a custom-built thermal gradient [18]. We also examined the influence of sex, age, and *Wolbachia* infection state on host thermal preference using the generalized linear model (GLM) analysis. We aimed to (1) investigate the role of *Wolbachia* in manipulating host thermal preference, and (2) explore its association with host age, sex, and population.

## 2. Materials and Methods

### 2.1. Leaf-Miner Collection and Rearing

**Figure 1:**
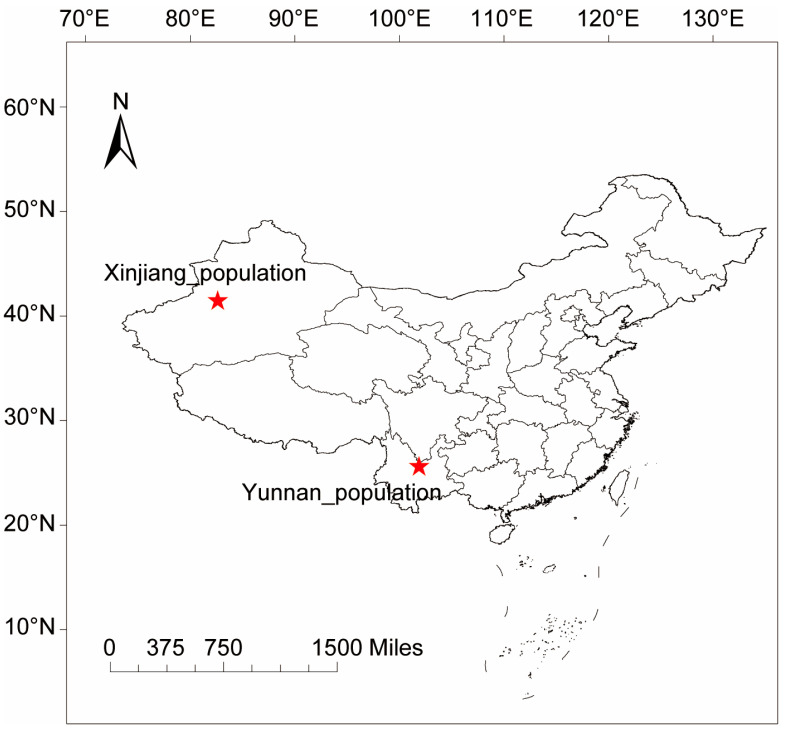
The sampling locations of the two leaf-miner populations. The template map, obtained from the Chinese National Basic Geographic Information Center (http://ngcc.sbsm.gov.cn) (accessed on 16 October 2020), was annotated using ArcGIS 10 Crack software.

To examine the effect of *Wolbachia* on the thermal preference of *L. huidobrensis*, we selected two representative populations of *L. huidobrensis*: Yunnan, where it was first reported in China, and Xinjiang, where it has recently spread after invasion [24,25,27]. These two wild populations, naturally infected with *Wolbachia*, were collected in March 2023 from cowpea plants in Yunnan (25.72° N, 101.87° E) and in April 2023 from tomatoes in Xinjiang (41.55° N, 82.62° E) (Figure 1). Upon transition to the laboratory, the *Wolbachia* wLhui-infected lines from each population were established. The wLhui-uninfected lines were derived through three generations of tetracycline treatment (1 mg/mL), followed by six generations of recovery without tetracycline to mitigate potential side effects. The *Wolbachia* infection status of each strain was verified via PCR before the experiment, as detailed previously [7]. All leaf-miner lines were maintained on bean seedlings under controlled laboratory conditions: 25 ± 1 °C temperature, 60% relative humidity, and a 16 h light/8 h dark cycle.

### 2.2. Temperature Preference Measurement

We employed a custom-built thermal gradient apparatus [18] to assess the Tp of the leaf miners. This apparatus consisted of a 50 cm long aluminum bar with a temperature gradient (ranging from 10 to 40 °C) generated by water baths positioned at each end. Six grooves were etched into the aluminum bar, enabling leaf miners to move freely along the gradient without interference. To prevent escape, these grooves were covered with a removable Plexiglas lid.

Temperature readings along the gradient were obtained using K-type thermocouples. All experiments were conducted in a room maintained at a constant temperature of 25 °C and 40% relative humidity. To collect data, six individuals from each strain were placed at the midpoint of each groove within the apparatus. After allowing 30 min for the miners to freely adjust within their respective grooves, the temperatures at their resting positions were recorded. At least seventy individuals from each group were assessed to determine the host temperature preference.

### 2.3. Statistical Analysis

All statistical analyses were performed and visualized using either GraphPad Prism version 9.00 or SPSS statistics version 20.0 for Windows (SPSS Inc., Chicago, IL, USA). We used the generalized linear models (GLMs) to assess the effects of *Wolbachia* infection status, sex, and age (1-day-old and 3-day-old) on the Tp of the leaf-miner host. The Tp of each individual was considered as the dependent variable, with age, sex, and *Wolbachia* infection status as fixed factors. The significance of the fixed factors and their interactions on Tp were determined. Additionally, the Mann–Whitney U test was used to compare Tp between wLhui-infected and uninfected hosts within each sex or age group. The Mann–Whitney test was also applied to assess differences in Tp between wLhui-infected and uninfected hosts in each population, irrespective of host age and sex.

## 3. Results

### 3.1. Characteristics of the Study Population

We assessed the thermal performance (Tp) of 1367 individuals from leaf-miner populations in Yunnan and Xinjiang (Figure 1). The Yunnan population included 642 individuals, comprising both 1-day-old and 3-day-old miners, with 321 *Wolbachia* wLhui-infected and 321 *Wolbachia* wLhui-uninfected individuals. The Xinjiang population consisted of 725 individuals, also split between 1-day-old and 3-day-old miners, with 368 *Wolbachia* wLhui-infected and 357 *Wolbachia* wLhui-uninfected individuals.

### 3.2. Wolbachia Infection Has No Effect on Tp in the Yunnan Leaf-Miner Population

**Figure 2:**
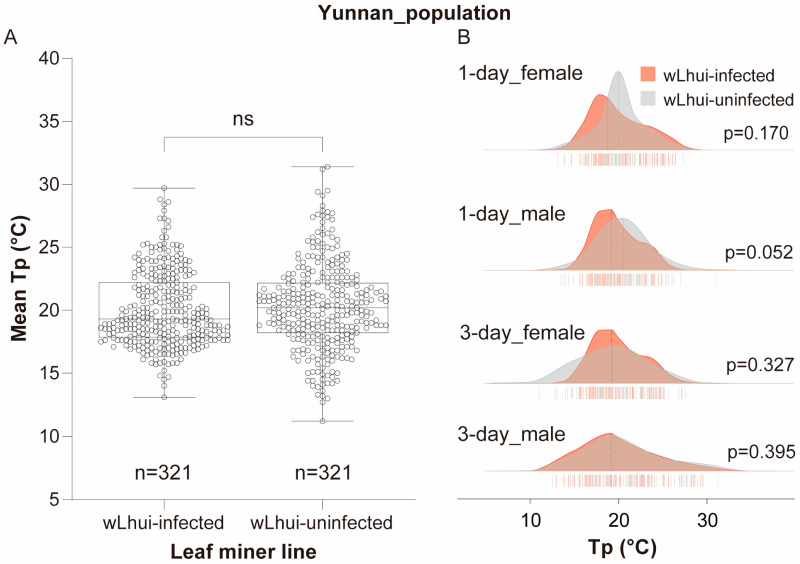
Thermal preference (Tp) of *Wolbachia* wLhui-infected and uninfected leaf miners in the Yunnan populations. (**A**) Boxplots illustrating Tp in the two leaf-miner lines without differentiating by host age and sex. The statistical significance between the two lines was assessed using the Mann–Whitney U test. ns, not significant. (**B**) Ridgeline plots depicting Tp in 1-day-old or 3-day-old female and male leaf miners, both infected and uninfected with wLhui. The Mann–Whitney test was used to assess significant differences between the two lines at the same age and sex.

When disregarding host age and sex, there was no significant difference in mean Tp between wLhui-infected and uninfected leaf miners in Yunnan populations (Mann–Whitney U test, *p* = 0.138; Figure 2A). The average Tp of wLhui-infected leaf miners (Tp = 19.30 °C) was slightly lower than that of wLhui-uninfected individuals (Tp = 20.20 °C), representing a difference of approximately 0.9 °C (Figure 2A).

When comparing the average Tp of wLhui-infected and uninfected leaf miners of the same age and sex, no significant differences were observed between the two lines (*p* > 0.05 for all cases; Figure 2B). Both lines of leaf miners exhibited a broad range of Tp values, spanning from 11.2 °C to 31.4 °C, with considerable overlap in the Tp distributions of the two leaf-miner lines (Figure 2B).

Furthermore, generalized linear model (GLM) analysis revealed no significant effects of *Wolbachia* (*χ*^2^ = 1.037, *p* = 0.308), age (*χ*^2^ = 0.444, *p* = 0.505), sex (*χ*^2^ = 1.918, *p* = 0.166), or their interactions (*p* > 0.05 for all cases) on host Tp (Table 1).

### 3.3. Wolbachia Infection Does Not Alter Tp in the Xinjiang Leaf-Miner Population

**Figure 3:**
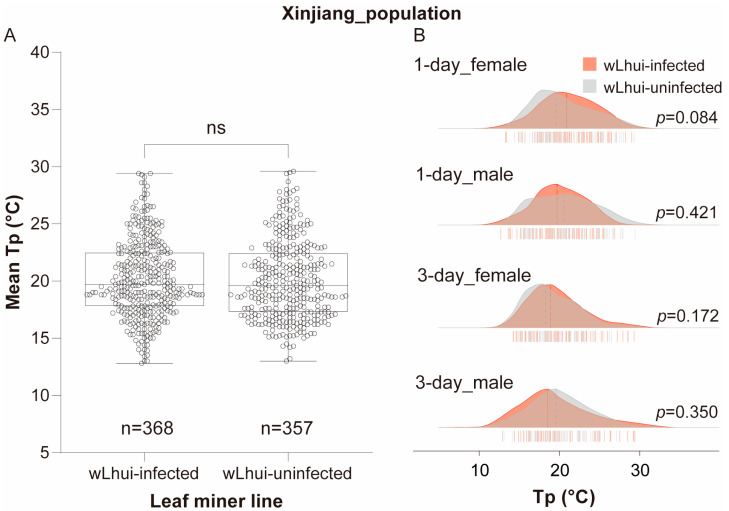
Thermal preference (Tp) of leaf miners infected with or without *Wolbachia* wLhui from the Xinjiang populations. (**A**) Boxplots illustrating Tp in wLhui-infected and uninfected leaf-miner lines, without considering host age or sex. Statistical significance between the two lines was assessed using the Mann–Whitney U test. ‘ns’ indicates no significant difference. (**B**) Ridgeline plots depicting Tp in 1-day-old or 3-day-old female and male leaf miners, infected or uninfected with wLhui, from the Xinjiang population. The Mann–Whitney U test was used to assess significant differences between the two lines at the same age and sex.

In the Xinjiang population, the average Tp values of wLhui-infected and uninfected leaf miners were nearly identical (wLhui-infected = 19.70 °C, wLhui-uninfected = 19.60 °C), with no significant statistical difference (Mann–Whitney U test, *p* = 0.469; Figure 3A). Similar results were observed when comparing infected and uninfected leaf miners of the same age and sex (*p* > 0.05 for all cases; Figure 3B). The Tp of the two leaf-miner lines in the Xinjiang population ranged from 12.8 °C to 29.6 °C, with some overlap in the Tp distributions (Figure 3B).

GLM analysis indicated that in the Xinjiang population, host Tp was strongly related to age (*χ*^2^ = 17.277, *p* < 0.001; Table 1). However, *Wolbachia* had no significant effect on host Tp, nor were there any interactions between *Wolbachia*, age, or sex (*p* > 0.05 for all cases; Table 1).

## 4. Discussion

The role of the endosymbiont *Wolbachia* in mediating the temperature preference (Tp) in insect hosts remains a topic of ongoing debate. In this study, we examined, for the first time, the relationship between the temperature preference of *L. huidobrensis* and *Wolbachia* infection under controlled laboratory conditions. Our findings provide no evidence that *Wolbachia* significantly affects the Tp of leaf miners in either the Yunnan or Xinjiang populations.

### 4.1. Divergent Influences of Wolbachia on Host Tp in Various Insect Species

Tp can vary significantly between populations of the same species [29,30]. In our study, we observed that Tp was strongly related to host age in the Xinjiang leaf-miner population but not in the Yunnan population, suggesting Tp may vary between these two populations. Nevertheless, we consistently found that *Wolbachia* had only subtle effects on Tp in both leaf-miner populations. Our results align with previous findings that show *Wolbachia* does not significantly impact host Tp [21], though they contradict other studies that report notable influences of *Wolbachia* on host Tp [16,17,19,20]. This suggests that *Wolbachia*-mediated host Tp can vary across different insect–endosymbiont systems.

It is widely recognized that the effect size and direction of *Wolbachia*-mediated host Tp are strongly influenced by several factors, including host background and genotypes, *Wolbachia* strains and titers, and environmental conditions [16,17]. Notably, Hague et al. [17] proposed that the effects of *Wolbachia* on Tp may exhibit a phylogenetic pattern; most *Drosophila* hosts infected with A-group *Wolbachia* strains prefer cooler temperatures, whereas species infected with divergent B-group *Wolbachia* strains prefer warmer temperatures, compared to uninfected genotypes [17]. Despite the fact that *Wolbachia* wLhui within the leaf-miner *L. huidobrensis* belongs to the A supergroup, we found no significant effect of *Wolbachia* on Tp. Therefore, the universal impact of A-group *Wolbachia* strains on insect host Tp remains uncertain and warrants further investigation. In addition to *Wolbachia* traits, the potential role of host traits and environmental factors in *Wolbachia*-mediated Tp in leaf miners are still unclear [16]. Additionally, the mechanisms underlying *Wolbachia*-induced changes in host Tp are largely unknown. Hague et al. [17] speculated that the differences in Tp between infected and uninfected insects might arise from the conflicting physiological requirements between *Wolbachia* and their hosts under temperature stress. The divergent influences of *Wolbachia* on host Tp may depend on specific co-evolutionary dynamics between *Wolbachia* and their hosts, resulting from trade-offs in thermal adaptation and balancing selection [18].

### 4.2. The Ecological Significance of Wolbachia-Mediated Host Tp

Small temperature fluctuations can significantly alter host–symbiont interactions [31,32]. These interactions, mediated by *Wolbachia*, play essential roles in host and *Wolbachia* ecology and evolution, including symbiosis maintenance, *Wolbachia* spread, and host adaptation to new environments [1,2,3,10,16]. For many invasive insects, *Wolbachia*-induced behavioral changes may confer a fitness advantage in novel environments, potentially accelerating population expansion and facilitating *Wolbachia*’s spread through host populations [28,33]. Given that we found no significant influence of *Wolbachia* on host Tp, we speculate that the rapid spread of *L. huidobrensis* may be not linked to *Wolbachia*-mediated thermal behavioral regulation. Instead, our recent work suggests that *Wolbachia* modifies host–cell metabolite profiles in response to short-term temperature stress, which may in turn affect the fitness and adaptive capacity of invasive *L. huidobrensis* [34].

In terms of practical applications, *Wolbachia* is utilized to control human diseases and agricultural pests through strategies such as the population replacement strategy (PRS) and the incompatible insect technique (IIT) [2,35]. Both strategies rely on the release of *Wolbachia*-infected individuals. Considering *Wolbachia*’s susceptibility to temperature [34], its potential positive regulation of host Tp in natural settings could mitigate the selection pressure from the thermal environment, thereby enhancing symbiosis stability and expanding its applications. Although we observed only subtle effects of *Wolbachia* on thermal preference, it could confer a fitness advantage in other ways, such as modifying host physiological responses to unsuitable temperature conditions [34], which could also enhance symbiosis stability. Given that *Wolbachia* induces complete cytoplasmic incompatibility in many leaf-miner species, there is great potential for using *Wolbachia*-induced incompatible insect techniques to control these pests [36,37].

Understanding *Wolbachia*’s potential role in influencing host thermal behavior is essential for unraveling the invasion dynamics of the leaf miner and lays the groundwork for future *Wolbachia*-based pest control strategies. Previous studies have shown that leaf miners often associate with multiple endosymbionts, with infection patterns that can vary spatially and temporally [26,38]. While this study focused on two representative populations of *L. huidobrensis* to examine the role of *Wolbachia* in host thermal behavior, distinct *Wolbachia* strains may exist in different populations [36], potentially influencing host responses to thermal changes. Nonetheless, our understanding of *Wolbachia*-mediated thermal preference in invasive leaf miners remains limited. Future studies should encompass a broader range of leaf miner–*Wolbachia* systems to explore the intricate interactions between *Wolbachia* and leaf-miner thermal behavior.

## Figures and Tables

**Table 1:** Generalized linear model (GLM) testing for the effects of host age, sex, *Wolbachia*, and their interaction on the thermal preference (Tp) in two leaf-miner populations.

Population	Factor	*χ* ^2^	*df*	*p*-Value
Yunnan	Age	0.444	1	0.505
	Sex	1.918	1	0.166
	*Wolbachia*	1.037	1	0.308
	Age × Sex	1.117	1	0.291
	Age × *Wolbachia*	1.032	1	0.310
	Sex × *Wolbachia*	2.499	1	0.114
	Age × Sex × *Wolbachia*	0.481	1	0.488
Xinjiang	Age	17.277	1	0.000
	Sex	0.012	1	0.911
	*Wolbachia*	0.242	1	0.622
	Age × Sex	3.299	1	0.069
	Age × *Wolbachia*	0.125	1	0.724
	Sex × *Wolbachia*	3.841	1	0.050
	Age × Sex × *Wolbachia*	0.177	1	0.674

## Data Availability

The data are provided in Table S1 of the electronic Supplementary Materials.

## Supplementary Materials

The following supporting information can be downloaded at: https://www.mdpi.com/article/10.3390/insects15100784/s1, Table S1: Thermal preference (Tp) of leaf miners infected with or without *Wolbachia*.
